# Rights, justice and climate resilience: lessons from fieldwork in urban Southeast Asia

**DOI:** 10.1177/09562478211035644

**Published:** 2021-08-22

**Authors:** Rebecca Mcmillan, Joanna Kocsis, Amrita Daniere

**Keywords:** climate change, governance, resilience, social learning, Southeast Asia

## Abstract

Recent transformative resilience research calls for urban climate interventions that better meet the needs of low-income and other marginalized groups. Such initiatives, it is suggested, must move beyond technocratic and superficial solutions to address the systems and structures that create climate vulnerability. While these are important theoretical developments, there is still much to be learned about how to support transformative resilience on the ground. This paper situates transformative resilience theory in practice with lessons from a five-year research partnership in Southeast Asian cities. We argue that for resilience research to advance rights and justice, knowledge production and mobilization efforts must be conceptualized as active parts of the transformation process. Bringing together conceptual and methodological insights from resilience, political ecology and governance learning research, we offer three pathways for transformative resilience and present examples of how they can be operationalized in Southeast Asia and beyond.

**Figure:**
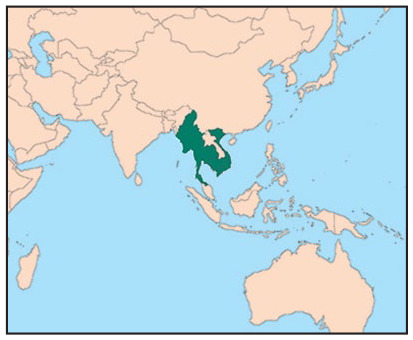


## I. Introduction

Building resilience to droughts, floods, extreme weather and other climate impacts is a major challenge for cities, particularly in the global South. Ziervogel et al. have invited us to rethink one-size-fits-all resilience approaches and begin building our efforts from *“locally situated processes, knowledges and norms”*.^(1)^ In particular, they call for a “transformative resilience” approach that goes beyond technological and infrastructural solutions towards addressing how institutions and economic development in different contexts can better support entitlements for the urban poor and other marginalized groups. Their work intersects with research on social learning for adaptive climate governance, which finds that transformational learning must involve greater participation in deliberative spaces where there is active reflection on, and effort to challenge, unequal power relationships.^(2)^ Together, this scholarship suggests that climate change adaptation should increase marginalized groups’ participation in governance, while advancing measures to address the inadequate water and sanitation, shelter, healthcare and insecure work that make urban life unsustainable for much of the world’s population. Focusing on rights and justice as core to transformative urban climate resilience is compelling and persuasive. Yet there is much more to be learned about how researchers and practitioners can support transformative resilience.

In this paper, we describe how, during five years of work in Southeast Asian cities, our project, the Urban Climate Resilience in Southeast Asia (UCRSEA) partnership,^(3)^ created opportunities for knowledge production and sharing that supported governance learning for resilience that foregrounds rights and justice. Based on these experiences, we argue that to operationalize theories of transformative resilience, the process of generating and mobilizing diverse forms of knowledge on everyday risks must be actively conceptualized and planned. Drawing on theoretical insights from political ecology and the principles of action research, we identified three ways that UCRSEA created the relevant opportunities for learning:

a) creating knowledge about everyday risks;
b) creating opportunities for producing and using diverse forms of knowledge to improve policy; and
c) using knowledge to engage and inform citizens.

While activities were tailored to our research context, it is our hope that these insights might be relevant to research work beyond Southeast Asia.

We begin with a brief review of the relevant literature and then introduce the readers to the five-year project (2014–2019). We subsequently discuss findings from the project’s knowledge production and mobilization efforts. We conclude with lessons for how researchers and practitioners might contribute to building transformative climate resilience.

## II. Literature Review: Learning Transformative Resilience

The term resilience is now ubiquitous in climate change planning for cities, and building a city’s resilience is widely seen as a key policy goal. While there are different understandings of resilience, it is usually defined as a system’s capacity to absorb shocks and respond to change in order to maintain its primary functions in spite of hazards and perturbations.^(4)^ These functions can be defined narrowly in terms of ecosystem services, or – as in the case of UCRSEA – expanded to include broader definitions of community wellbeing.^(5)^

Resilience thinking originates in research on social-ecological systems (SES). SES is a branch of ecology that explores the dynamic interactions between different components of complex adaptive systems such as ecosystems, recognizing them as coupled human and environmental systems. While SES work was initially concerned with protected areas or less populated environments, its insights have been adapted to urban areas. Building on SES’s holistic approach, urban resilience work sees cities as complex “systems” composed of multiple ecological, technological and institutional subsystems. Proponents argue that SES usefully highlights the dependencies between urban systems and networks within and outside of the immediate area of a city – stressing, for example, the reliance of urban systems on interconnected water, transport and electricity infrastructures, and the institutional systems that govern them and shape rights and entitlements.^(6)^

Climate change impacts, in turn, cascade across these different urban subsystems, with compounding impacts for human and environmental systems.^(7)^ Given the unpredictable and unprecedented nature of urban climate change, the resilience literature suggests the need to move beyond conventional predict-and-plan approaches^(8)^ to more adaptive, flexible and participatory governance arrangements that help actors learn and respond to change.^(9)^

While drawing attention to these complex interlinkages, urban resilience approaches – concerned with the system as a whole – are less equipped to consider how issues of power and resource access produce differential vulnerability within an urban environment, and between cities and other scales of action.^(10)^ Political ecologists and critical development scholars take up these vulnerability concerns, emphasizing different groups’ exposure to risk, as well as the diverse forms of knowledge, entitlements and relationships that enable them to adapt to hazards and change.^(11)^ Given the uneven distribution of these capacities, and the fact that security for some can come at the expense of the security of others’,^(12)^ critical observers call for conceptual and policy clarity around “resilience of what?” and “for whom?”^(13)^

Critical resilience scholars suggest that resilience policy is overly technocratic, focused on “climate-proofing” urban infrastructures.^(14)^ In defending the status quo, such approaches can overlook the way urban governance and development systematically marginalize particular social groups, even in the absence of climate change impacts.^(15)^ Resilience approaches that fail to challenge inequity are, at best, ineffective in protecting vulnerable groups and, at worst, can exacerbate inequality, downscaling responsibility for confronting climate change to marginalized citizens with the least capacity to cope.^(16)^

Responding to these different framings of resilience, Pelling^(17)^ identifies different “levels” of adaptation practice as: resilience (preserving the existing system), transition (working around the edges of the existing system to improve conditions), and transformation. Transformative approaches go beyond attenuating the adverse effects for prevailing systems, towards seeking to transform underlying structures and processes to enact equitable climate futures.^(18)^ As Ziervogel et al. highlight, the roots of vulnerability often lie in urban dwellers’ lack of *entitlements* to such basic needs as water, sanitation and housing, which are in turn shaped by institutional failures and political economic processes driving urbanization. Focusing on these basic rights, they suggest, *“. . . may help to address many of the root causes that characterize the unacceptable risks that urban residents face on a daily basis”*.^(19)^

The transformative resilience approach pushes researchers, practitioners and policymakers to engage more explicitly with questions of fairness and justice^(20)^ by supporting institutions and processes that challenge unequal power relations, empower marginalized groups, and distribute the benefits and burdens of urbanization more equally.^(21)^ This requires that researchers and decision makers engage more directly with marginalized groups in their work,^(22)^ a fundamental paradigm change in thinking and practice for many.^(23)^ In the language of systems theory, it requires a shift to triple-loop learning, critically examining the values and norms undergirding existing systems and processes to achieve fundamental transformations in governance, not just improvements in current models.^(24)^

Scholarship on risk awareness, communication and knowledge co-production dovetails with the transformative approach, and can provide insights on operationalizing learning leading to broader transformative change. Social learning helps identify and correct ineffective public policy and practice, but can also support social change more broadly.^(25)^ Specifically, when institutions, organizations, communities and other groups engage in triple-loop learning, they are driven to question existing practices and underlying societal norms and values. This can lead to entirely new ways of allocating resources and making decisions. Providing opportunities for a range of local stakeholders to engage in shared experiences and learning opportunities around a collective crisis or common concern is crucial to this transformation process. However, these efforts, according to Wolfram and co-authors, need *“to be situated, stressing the importance of context, personal exchange and learning-by-doing as critical conditions”*.^(26)^

While we believe that calls for transformative resilience research and triple-loop learning are entirely justified, there is much to be learned about fostering them in practice, particularly in the relatively closed political contexts of many Southeast Asian countries. This gap reflects a broader dearth of relevant on-the-ground analyses of transformative adaptation and resilience practices.^(27)^ This paper contributes evidence on how researchers and planners can actively support transformative resilience processes drawn from the experience of the UCRSEA project.

## III. Context and Methodology

**Map 1:**
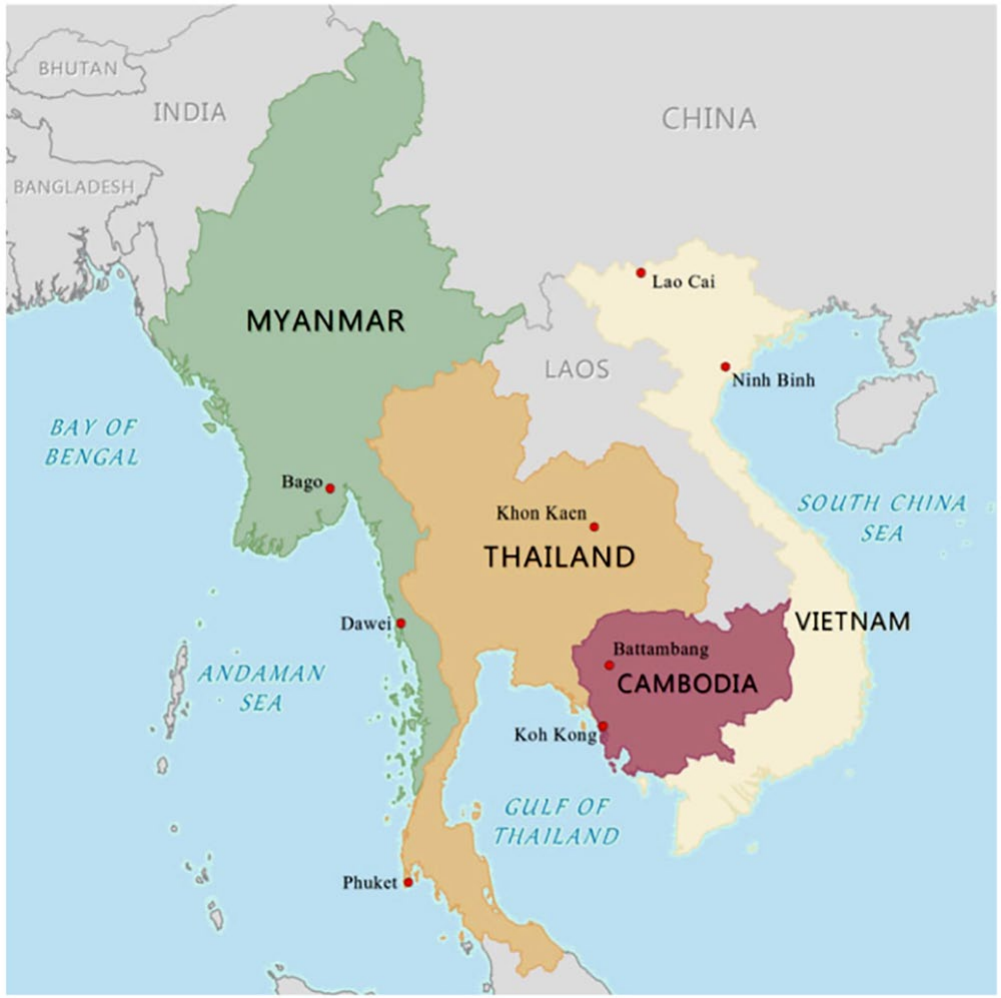
UCRSEA case cities NOTE: Made with Natural Earth.

The UCRSEA project was designed to address growing everyday risks posed by climate change in cities within Southeast Asia. Throughout the region, urban expansion in coastal areas, deltas and river basins has intensified climate-related vulnerabilities in already ecologically fragile areas.^(28)^ Given evidence from the region that climate change disproportionately affects those on low incomes, exacerbating existing socioeconomic inequalities,^(29)^ UCRSEA sought to support the adaptation of the most marginalized groups. UCRSEA researchers carried out fieldwork in secondary cities in Cambodia, Myanmar, Thailand and Vietnam (Map 1 and Table 1), all experiencing dramatic urban and economic growth following periods of conflict.^(30)^ The partnership brought together multiple organizations with diverse backgrounds and expertise in these cities to conduct research and build resilience capacity (see the online supplementary information).

**Table 1:** UCRSEA case cities and research themes

Case City	Research Focus (as it intersects with climate change)
Battambang, Cambodia	Disaster risk governance; land rights, housing, and community resistance
Koh Kong, Cambodia	Disaster risk governance; migration and wellbeing in fishing communities
Bago, Myanmar	Flood risk management and governance
Dawei, Myanmar	Special economic zone development, livelihoods and risk; water supply and access in informal settlements
Khon Kaen, Thailand	Flood risk and livelihoods in peri-urban areas; water supply and access in informal settlements
Mukdahan, Thailand	Special economic zone development, livelihoods and risk
Phuket*, Thailand	Migration, tourism and livelihoods
Lao Cai, Vietnam	Local implementation of climate change policy and plans
Ninh Binh, Vietnam	Ecotourism, gender and livelihoods; industrial zone development and water-related risk

* NOTE: Phuket was not an official case city, though UCRSEA supported doctoral research there.

Governments in Southeast Asia have been unsuccessful in building climate change resilience, and especially in managing natural disasters, because of poor coordination, a lack of monitoring and evaluation, rigidity, poor transparency, and the domination and corruption of community-level planning and governance by well-connected elites.^(31)^ These are compounded by the “wicked”^(32)^ nature of climate change, urbanization and everyday risk, characterized by what Turnbull and Hoppe call *“persistently problematic distances between stakeholders”*.^(33)^ This is particularly true in urban areas, given ethnic and class diversity, and the exclusion of marginalized groups from governance processes. New mechanisms of collective decision making and promoting state–civil society linkages to advance rights and justice thus remain pressing research concerns.

To begin addressing this research gap, UCRSEA sought to build knowledge of everyday risks and identify ways to address them through practical actions. Underpinned by action research principles, UCRSEA activities included not only data collection, but also knowledge mobilization, training and partnership development to build local capacities for resilience. UCRSEA partners were encouraged to use action research methodologies when possible, combining knowledge production and stakeholder engagement with concrete interventions to achieve desired ends through iterative cycles of action, learning and reflection.^(34)^ The project’s multi-pronged research framework conceptualized research and action as integrated, iterative processes.^(35)^

We further sought to support triple-loop learning^(36)^ by building from researchers’, policymakers’ and citizens’ existing norms and practices to establish a collective framing of resilience that privileged rights and justice. Rather than taking for granted that all urban stakeholders share a vision of the urban system and what resilience means (for a critique see Reed et al.^(37)^), we see resilience as a *negotiated* process.^(38)^ Informed by the collective impact approach,^(39)^ UCRSEA partners were committed to creating spaces for researchers and stakeholders to frame contentious urban governance problems and conceive of solutions together, i.e. to “co-produce” knowledge.

In this approach, all governance actors, including researchers, community members and policymakers, could learn about complex urban resilience issues from multiple perspectives. This encouraged the integration of knowledge from diverse sources, key to achieving the depth of learning required for transforming entrenched beliefs or paradigms.^(40)^ Multistakeholder participation is recognized as key to climate-resilient governance, given that learning and adaptation in complex systems require feedback from multiple sources with different knowledge of the system or its subsystems.^(41)^ This collaboration can bring governance processes better in line with multiscalar, interconnected environmental problems in cities.^(42)^

In UCRSEA, an important tool for linking research, dialogue and action is the theory of change (ToC), a shared conceptual map of the relevant systems, actors and processes, which helps researchers envision the levers for change they might engage in producing and mobilizing knowledge. Theory of change thinking can, according to Vogel, *“create a strong organizing framework to improve programme design, implementation, evaluation and learning”*.^(43)^ It has been applied with success in the fields of international and community development and evaluation.^(44)^ For recent examples of ToC use in complex cases see Derbyshire, Guarneros-Meza et al. and Rolfe.^(45)^ During collaborative workshops, UCRSEA members developed a ToC for the network as a whole, as well as specific ToCs for each country partner. At both the network and project levels, the ToCs identified the mechanisms through which objectives might be achieved, different actors’ contributions, and the logics behind each activity. They also facilitated enhanced understanding of how governance actors learn^(46)^ throughout the five-year process by encouraging ongoing reflection on, and adjustment of, activities and objectives to better address the learning needs of each group.

The UCRSEA partnership-level ToC prioritized the project’s overall objective: that *“communities in Southeast Asia become more socially, ecologically and economically resilient to climate change”*, an achievement that enhances *“possibilities for green growth, better governance, and greater security and sustainability”*.^(47)^ In a series of workshops, partners broke down the actual steps needed to accomplish this outcome at both the network and city levels – changes in knowledge, in skills, in political will, and in access to decision making. Each research partner organization in the eight case cities also carried out research projects that supported the partnership-level ToC. In addition, they relied on their own project-level ToCs to frame work on everyday risks in their case cities – from industrial zone development in Mukdahan, Thailand and Ninh Binh, Vietnam, to peri-urban flooding in Khon Kaen, Thailand (Table 1). The ToCs helped researchers position their efforts to engage and influence key stakeholders from the private sector, government, and local communities of interest.^(48)^

In the remainder of the paper, we outline lessons that emerged from the research and knowledge mobilization processes. We identify three key actions or entry points for transformative resilience: (1) producing knowledge about everyday risks; (2) using knowledge to influence policy change; and (3) using knowledge to motivate and inform the average citizen. While addressed here sequentially, the three processes were carried out in tandem. For example, research emphasized the co-production of knowledge through policymaker and community engagement. We have chosen a small number of illustrative examples from our work to clarify how various stakeholders can insert rights and justice more transparently into climate resilience. Examples were selected to showcase at least one project from each UCRSEA country of focus and to describe a range of research initiatives. Given space limitations, the cited examples do not fully represent the empirical richness of partners’ research findings, nor the breadth of research and engagement in each city. Case studies are more fully documented elsewhere.^(49)^

## IV. Knowledge Creation And Mobilization

### a. Producing knowledge on everyday risks

Knowledge-production activities were designed with a deliberate focus on working with local, marginalized groups to better understand their vulnerability and adaptive capacity in the face of everyday risks. UCRSEA researchers drew on concepts from systems approaches to resilience, political ecology and actor-oriented livelihoods approaches. While systems approaches (discussed above) draw attention to interconnected social, technical and infrastructural systems, political ecology deliberately acknowledges the power-laden political, economic and governance dynamics shaping those systems and creating winners and losers. The vulnerability of different groups to change, for example, is shaped by the institutions and processes that mediate households’ and communities’ access to entitlements. Actor-oriented approaches centre the voices and agency of these different stakeholders: they combine assessments of different groups’ adaptive capacities, and explore how they make meaning of their situations and work to change them.^(50)^

To develop a joint understanding of vulnerabilities in their case cities, all UCRSEA project teams conducted vulnerability assessments. These sought to better understand the combined impacts of climate change and urbanization on specific marginalized groups, and explored the capacity of governments and communities to respond to change. Researchers adapted internationally recognized methodologies for climate vulnerability assessment and disaster risk reduction analysis, including sustainable livelihoods assessments^(51)^ and the UN Office for Disaster Risk Reduction (UNISDR) Disaster Resilience Scorecard for Cities,^(52)^ to their local contexts. They carried out the assessments in cooperation with local civil society and policy stakeholders. This helped them develop common conceptual framings of resilience and vulnerability from which to then devise concrete policy and intervention proposals. Through engagements with the regional UCRSEA network, researchers were continuously relating the local and national dynamics in their case cities to broader transformative processes, illustrating the links between community vulnerability and global and regional economic integration.

Research carried out in Khon Kaen, Thailand (Map 1) illustrates the connections among climate change, political and economic processes, and everyday climate risks. Khon Kaen is a rapidly growing secondary city in Northern Thailand and an important economic and logistical hub connecting Thailand to the region and China.^(53)^ The city faces impacts from global climate change, including both increased flooding from growing rainfall intensity^(54)^ and drought periods.^(55)^ UCRSEA researchers from Mahasarakham University (MSU) sought to better understand how residents in Khon Kaen respond to flooding, by focusing on the adjacent low-lying peri-urban municipality of Pra Lab.^(56)^ Already squeezed by development, Pra Lab’s mainly agricultural and aquaculture-based livelihoods are further threatened by regular flooding due to water runoff from higher municipalities, as well as overflow from the Chi and Phong Rivers that border the settlement.

MSU researchers drew on UCRSEA’s Climate Resilience Framework to focus on ecological, political, economic and infrastructural dynamics in Khon Kaen.^(57)^ This was combined with a livelihood vulnerability index (LVI) based on surveys with 236 Pra Lab households. The index explores five types of assets that support residents’ climate adaptation: human, social, natural, physical and financial. It also measures the vulnerability of Pra Lab residents in relation to shocks, stress and seasonality. With attention to the agency of Pra Lab residents, the LVI focuses on the strength of community livelihoods and their adaptive strategies.

Crucially, the researchers’ multiscalar analysis revealed that flood risk is systemic: built into urban planning and governance processes in the local area dominated by Khon Kaen.^(58)^ In the Khon Kaen region, suspension of land use regulations, for a decade or more, enabled vast land conversions from floodplains to functions related to urban and industrial growth. From a systems-inspired resilience perspective, the urban ecosystems that support urbanization are being undermined as impervious surfaces increase runoff and as development blocks natural floodways. This is exacerbated by poor drainage. The political ecology perspective highlights the creation by the urban development process of specific winners and losers, since floodgates protect the city centre while diverting water to surrounding areas. Pra Lab, as a low-lying agricultural settlement on what was traditionally a floodplain, is particularly vulnerable. Unsurprisingly, its marginalized residents lack political voice in municipal decision-making processes.

The conventional hazards-focused approach that tends to dominate planning in Thailand and elsewhere in Southeast Asia^(59)^ would likely seek to solve this problem through technical means, such as additional floodwalls and floodgates. However, MSU researchers’ assessment suggests that this approach – unaccompanied by planning reform – would simply divert the problem elsewhere, failing to address the root causes of vulnerability that lie in processes of urbanization and planning. Moreover, better floodgate management relies on coordination of the many agencies involved, which is currently lacking. In contrast to “hard” infrastructure solutions, reducing vulnerability for Pra Lab residents requires “soft” solutions such as support for livelihood transitions given declining agricultural viability, and changes to land use planning and water management priorities. The researchers presented their findings to Khon Kaen’s City Municipality (members of whom had participated in the research itself) and encouraged policymakers to reframe the problem and its solutions to embrace a more rights and governance-based approach. Pra Lab residents were involved in the presentation.

Research in Khon Kaen also highlights the importance of attention to *everyday* forms of risk, or what Ziervogel et al. call the *“slow and ongoing catastrophe of the failings of everyday development”*,^(60)^ going beyond the more catastrophic disaster events that tend to grab media and policy attention. This was a methodological challenge since the official historical data only included disaster events. Qualitative interviews with local residents – a key element of the actor-oriented livelihoods approach – revealed that flooding was more regular than these records suggested, and frequently impaired local livelihoods.^(61)^ Regular flooding preceding harvest periods caused severe crop losses, putting pressure on residents’ already precarious agricultural incomes, and propelling some into a spiral of debt. This also occurred in other research settings, such as in Dawei, Myanmar.^(62)^

The need to learn from urban residents about how climate change and urbanization exacerbate their everyday risks emerged in nearly all of the UCRSEA researchers’ work. Research in Ninh Binh, Vietnam, for example, adds a new dimension to the analysis: it illustrates how efforts in the name of “green growth” that might increase the resilience of the urban system as a whole involve trade-offs and can undermine security for some residents. Ninh Binh, located in the southern portion of the Red River Delta about 100 kilometres southeast of Hanoi, in Northern Vietnam (Map 1), is slated to become a level 1 urban centre by 2050^(63)^ and a world-class “green” tourism destination by 2030. Two research teams worked in Ninh Binh, focusing on industrialization and ecotourism respectively. Researchers from the Center for Environment and Community Research (CECR) investigated the Trang An Landscape Complex (TALC), recognized by UNESCO as a World Heritage Site for its natural and historical significance. In a first for tourism in Vietnam, TALC is led through a public–private partnership model. *“Tourism development has already prompted the area to urbanize rapidly, transforming physical landscapes and livelihoods.”*^(64)^

To understand how Ninh Binh residents are weathering the changes associated with ecotourism, climate and livelihood change, CECR’s team conducted a detailed assessment of local livelihoods and wellbeing in the Truong Yen commune. With the creation of the tourism complex, 90 per cent of the commune’s land was converted from agriculture to tourism, and communal land use rights were transferred to a private tourism company.^(65)^ Researchers also examined existing social protection measures among communities, as well as government preparedness. To assess the latter, they employed the UNISDR participatory Disaster Resilience Scorecard for Cities.^(66)^ While researchers found that ecotourism was improving incomes and providing some climate resilience benefits, they also noted that urbanization was creating new forms of insecurity and reshaping local power relationships in ways that undermined residents’ livelihood security. Today, people’s access to basic entitlements, such as water and waste management, are increasingly threatened, creating new burdens that fall heavily on women.

As agriculture becomes less feasible due to land loss, many residents work at least part-time in the tourism sector and some are now more dependent on these cash incomes than on subsistence farming. For example, 2,600 women are now employed as boat riders (who both steer the boat and provide information) who take visitors along scenic routes through the caves.^(67)^ While this reduces their vulnerability to climate risks in some ways, it also makes them increasingly reliant on cash to secure their basic entitlements, such as food. Wages in the tourism sector are low and competition is high. Tourism is not as climate sensitive as agriculture, but access to the scenic routes depends on water levels, so extreme conditions associated with climate change can interrupt much-needed income. As the public–private partnership gains authority, lines of accountability are increasingly complex and blurry, and local people frequently find that their grievances fall on deaf ears. As in Khon Kaen, this case illustrates how climate resilience requires attention to livelihood supports and the right to participate in governance, as much as protection from hazards. Hoang and Pulliat^(68)^ argue that training local people (especially youth and women) to better harness the benefits of ecotourism would reduce climate vulnerability while also strengthening longer-term resilience of both the community and the tourism economy.

Bringing together a multiscalar analysis of vulnerability – combining a systems focus on resilience with an actor-oriented emphasis on people’s agency – with political ecology’s attention to broader political and economic power relations, helps draw attention to questions of governance and, in particular, the need to guarantee people’s entitlements in the context of change. While reframing climate resilience in terms of governance and entitlements might make these problems seem intractable, in the next subsections we show that building dialogues around everyday risk can help create networks of interested actors, and provide a platform for addressing larger structural issues.

### b. Using knowledge to engage policymakers

When partners linked climate change resilience to the everyday risks affecting local actors’ lives, they could identify concrete needs that policymakers could work to address. This lens encouraged collaboration between previously siloed groups, like different government departments, policymakers, researchers and civil society members. This collaboration is important given the tendency for climate change to be relegated to environmental departments in Southeast Asia, rather than “mainstreamed” as a systemic issue. Responding to the resilience literature’s call for adaptive, problem-oriented, and experimental or iterative policymaking^(69)^ poses other particular challenges. For one, governance norms in the four countries studied emphasize rigid, top-down and centralized governance structures.^(70)^ Meanwhile, centralization or incomplete decentralization in some Southeast Asian countries^(71)^ means cities often lack the necessary capacity for addressing complex urban climate challenges.^(72)^ This “capability trap”^(73)^ is especially pronounced in secondary cities.^(74)^ Indeed, UCRSEA researchers identified serious barriers to adaptive and resilient governance in their case cities.^(75)^

Fostering inclusive governance that addresses everyday risks thus demands an innovative approach to governance learning that works within these constraints, and builds on the daily practices of policymakers and the opportunities available to them to enact change.^(76)^ UCRSEA’s policy engagement work brought researchers, policymakers and other actors together in regular and iteratively structured social learning activities. At these events, researchers who work closely with communities brought knowledge about everyday risks; policymakers brought knowledge about governance; and the UCRSEA team brought skills and tools to support social learning. These events allowed two pathways for learning that enhanced the UCRSEA partners’ work: (1) connecting researchers with policymakers early in the research process, and iteratively throughout it, to produce new knowledge about climate vulnerabilities and the role of policymakers in addressing them; and (2) building momentum for triple-loop learning through activities that developed a shared framing of resilience. Work along these pathways helped to reframe policymakers’ thinking away from the hazards focus that has dominated regional planning, towards recognizing the importance of everyday risk and inclusive development.

Central to UCRSEA stakeholder engagement and its theory of change were tools from outcome mapping (OM), a methodology used to plan, monitor and evaluate development projects with a view to sustainable social change.^(77)^ When used in the planning stage of a project, the OM process allows project teams to be specific about the actors they intend to target, the changes they hope to see, and the strategies they might employ to achieve their goals.^(78)^ In our ToC workshops, participants created a vision of the future to which their work would contribute by describing what the community would look like if their vision were achieved.

Next, we used the ToC to help us map backwards to identify the necessary changes to achieve that vision. Partners were able to identify the boundary partners (BPs) – individuals and institutions whose behaviour needed to change to achieve the vision – and discussed how researchers might influence this change by reframing resilience issues, developing skills, generating evidence or connecting them with other stakeholders. Recognizing that many of the requisite structural changes lie beyond the scope of researchers’ capacities, the “sphere of influence” concept was useful in determining where our research could have a tangible impact on everyday risks by identifying other stakeholders whose behaviour we *could* influence. In individual projects, researchers sought to engage BPs early in the research process to ensure that we understood their information needs, and that the research would be useful for them.^(79)^ In this way, UCRSEA engaged a range of key governance actors, including researchers, policymakers and community stakeholders, in the learning process.

At the network level, UCRSEA supported researchers to engage with these BPs by leveraging policy contacts to secure one-on-one meetings and inviting policymakers to join our semi-annual city visits or annual research workshops. During our annual workshop in Battambang in May 2017, Cambodian municipal and national policymakers were invited to address the partners to help UCRSEA researchers better understand their tasks and accountability structures. In this interactive session, researchers and policymakers discussed policy windows of opportunity, standards of evidence for policymaking, and the technical and political challenges of moving from knowledge to policy. In another session at the same workshop, the four members of our international advisory board (IAB) (including the former director of the Asian Disaster Preparedness Center, the deputy director of the Research Centre of Science and Technology Policy of Vietnam, and the former UN assistant secretary-general of the UNEP and UNDP – Asia Pacific Regions) shared their experiences of how transformative change takes place within global institutions.

The UCRSEA process brought together actors from different communities (“exogenous” actors^(80)^) to create opportunities for governance learning based on diverse and relevant forms of knowledge. The insights of policymakers and IAB members helped researchers understand pathways for research uptake and systems change. At the same time, policymakers and IAB members learned about everyday risks facing communities in more qualitative depth than they would have through typical research–policy communication channels. Workshops were iterative and frequent, allowing participants to establish closer and more informal relationships through learning in “parallel”^(81)^ or “shadow spaces”.^(82)^ Shadow spaces are seen as beneficial for triple-loop learning for transformative change since they can promote creativity and critical thinking, free of the constraints of bureaucratic norms and procedures. Learning in shadow spaces can, in turn, spill over and influence practices and norms in formal institutions.

UCRSEA tracked the changing needs of its governance partners throughout the project and designed opportunities for governance learning as relationships and contexts evolved. This included providing researchers with extensive policy influence and knowledge mobilization training, and creating opportunities for researchers, policymakers and civil society members from across the Greater Mekong Subregion to come together to learn new concepts, visit research sites, and exchange knowledge and experiences.

The project’s final event, a policy forum in Bangkok in 2019, brought together researchers and high-ranking policymakers from across the region to encourage collective agenda setting to prioritize urban climate resilience. One particularly useful learning tool at that event was the applied learning simulation. Using a fictional case, stakeholders engaged in a role-playing activity in which participants had to represent the interests of a group with quite different experiences and stakes than their own. This activity built knowledge, skills and intergroup empathy in ways that traditional researcher–policymaker interactions cannot, while implicitly drawing on concepts from political ecology.

While triple-loop learning to change institutional norms and policymaking for transformative resilience is a long process, policymaker participants in the network’s activities reported seeing problems in new ways, indicating they had benefitted from double-loop learning or changes in “problem framing”.^(83)^ Perhaps more importantly, these activities have fostered the creation of policy–civil society–researcher networks that have outlasted UCRSEA’s five-year lifespan. By linking climate change resilience to everyday risks in practical ways, researchers created spaces in which policymakers, researchers and civil society members could work towards shared objectives based on common understandings.

One important change related to the work of the UCRSEA partnership to date is the impending establishment of the Urban Planning and Development programme within the Faculty of Development Studies at the Royal University of Phnom Penh (RUPP) in Cambodia. This initiative has been largely driven by UCRSEA partners from RUPP who identified a need to enhance the faculty’s ability to address development issues by adding social science-based teaching and research into a department thus far focused primarily on natural science and engineering.^(84)^ The design of this four-year programme is largely informed by UCRSEA’s Urban Climate Resilience Curriculum, which introduces university students to the concepts and tools of urban resilience thinking, and the knowledge created through the participation of RUPP faculty and students, as well as key governance actors from the Cambodian Ministry of Environment, in UCRSEA’s iterative learning activities. The creation of a university programme based on UCRSEA’s conceptual framework demonstrates triple-loop learning among agenda setters in the university, the Ministry of Environment, and the Ministry of Education, Youth and Sport. The establishment of this programme demonstrates a shift in the perception of urban climate resilience issues and urban planning priorities within Cambodia. Will also train new generation of policy and research leaders – another main pillar of UCRSEA’s work.

### c. Using knowledge to engage civil society

The above discussion suggests that, while there is some political will and interest in policies that advance rights and justice for more transformative resilience, there is still a long way to go. Given the closed political regimes in many Southeast Asian countries, as well as the lack of resources and capacity in secondary cities, it is not realistic to expect adaptive and inclusive governance to emerge in these cities without both external support and local social pressure. Yet there are scant studies on the possible emergence of transformative and participatory planning practice in these top-down, centralized political environments.^(85)^ The UCRSEA project recognized the key role that civil society and citizen knowledge development – through citizen science and participatory research – can and should play in implementing climate change adaptation measures, and sought to better understand ways to do this in practice. One way was using knowledge production activities as an opportunity to engage and inform citizens.

Based on her research on collective action in informal settlements in Jakarta, Beard^(86)^ notes that social learning for transformative change will tend to be incremental in rigid political contexts. Conversely, in repressive contexts, citizen resistance may take subtle, apparently mundane or covert form. These acts can develop social capital and the capacity for deeper transformation when a political window of opportunity opens.^(87)^ Beard’s research suggests that creating opportunities for civil society actors to learn about climate change, and the potential for building resilience in ways that work to empower them, may allow cities to implement change in an incremental and, eventually, more effective way. Within the UCRSEA project, some of the researchers embraced methods that engaged citizens directly through participatory knowledge creation, in line with action research principles that seek to democratize the research process by challenging the separation of layperson and expert.^(88)^ This, in turn, can raise consciousness and provide a springboard into other forms of political action.

A compelling example from the UCRSEA project was the project’s AirBeam initiative. The AirBeam, a palm-sized unit developed by a company called Habitatmap, allows users to measure air quality, including temperature, humidity and particulate matter, in their immediate vicinity. The units are remarkably accurate and cost a few hundred US dollars apiece. Once data are collected at a particular location, they can be uploaded immediately to an open global website and shared with all other AirBeam users, providing a real-time international opportunity to compare and learn with other similarly motivated citizen scientists. The UCRSEA project invested early on in eight AirBeams and distributed them to our research partners to engage citizens in urban climate and environmental research, and create new platforms for their voices to be heard.

Partners deployed the AirBeams in a variety of ways adapted to their local contexts. In Hanoi, Vietnam, the AirBeams were used in primary and secondary schools to teach schoolchildren and teachers how to use the device to collect information about local urban air quality. A secondary school science teacher affiliated with the project borrowed an AirBeam from the Centre for Environment and Community Research (CECR) and devoted an entire curriculum unit to the measurement and comparison of air quality in different parts of the city at different times, as well as comparing it with other cities, to help his students learn about pollution and climate change. In addition, CECR experimented with sharing the AirBeam technology as part of Earth Day 2017 celebrations in a major park in Hanoi.^(89)^ CECR claim that the AirBeam has become one of their most important ways to engage and inform people in Hanoi about the importance of learning about and adapting to climate change.

In Myanmar, two of the research partners travelled to various cities where they trained university faculty and high school teachers in collecting data with AirBeam, especially for measuring Particulate Matter 2.5 (PM 2.5).^(90)^ The participants travelled around the city and collected data to upload to the AirBeam website. The workshops were well attended across the entire country. In Dawei, a coastal city located in the south of the country close to the border with Thailand, attendees found real-time PM 2.5 air quality in the northern part of the city to be in the range of 55.5 to 150.4 (μg/m^3^). According to the Air Quality Index standards, this is just shy of the unhealthy range (151 to 200 μg/m^3^), which shocked many workshop participants, given the city’s small size and relative lack of development.

The enthusiasm of the individuals involved, many of them students, was documented by the partners through photos and short videos, as well as surveys administered to students before and after they used the AirBeams.^(91)^ Social scientists have long acknowledged the value of environmental knowledge for motivating individuals to learn more and participate in shaping the future (for a recent example, see Liobikien and Poškus^(92)^). It is clear that this kind of citizen science conducted with children and teenagers offers an untapped space to raise climate change awareness and encourage users to consider what might be done to decrease their vulnerability to heat, drought and pollution in the future. Starting from everyday risks – like air pollution – can open conversations about larger structural issues, such as climate change policy and practice. One recommendation from this experience is that widespread introduction of a user-friendly tool, even in resource-constrained environments, has transformative power. The use of these devices can be supported by local governments or NGOs, and can empower citizens to engage with national authorities around climate change impacts and potential adaptations.

Compared with Vietnam, where climate change information is shared quite liberally and has become a national policy priority, in Myanmar it is much less of a focus – the result of the country’s recent violent political history and precarious economic circumstances.^(93)^ The national government there has not prioritized climate change policy and planning to any significant degree, showing the high relevance of the UCRSEA-generated evidence that knowledge can empower local actors to engage at other scales. The lesson here is that knowledge production can be used to engage citizens of all ages by motivating and empowering them to take an interest and stake a claim in the decision-making processes that affect their lives.

## V. Discussion

All of these cases, and others from the UCRSEA work,^(94)^ support Ziervogel et al.’s^(95)^ argument that resilience work needs to foreground rights, justice and entitlements. In research, this must begin with a better understanding of the governance failures and political and economic processes that shape access to entitlements. One key lesson from UCRSEA’s knowledge production work is the value of linking systems and political ecology perspectives with a more fine-grained analysis of local vulnerability and agency. Localized vulnerability assessments highlight how people’s capacity to absorb and respond to shocks is shaped by unequal entitlements. However, focusing only on the local level can make it difficult to understand the national, regional and global drivers of change that undermine entitlements, and the forms they will take in the future. All UCRSEA projects identified governance as a key challenge and found that leveraging everyday risks could bring together different actors to support dialogue around climate resilience and vulnerability.

UCRSEA work suggests that researchers can enhance the effectiveness of local governance by acting as knowledge brokers and fostering the creation of networks across the state–society divide. To date, there has been a persistent disconnect between academic knowledge production on the effects of climate change, the utility of such knowledge for policy, and decision makers’ capacities to apply such knowledge to generate change.^(96)^ We see promising efforts to address this through more participatory research processes, but even where the data collection process is participatory, knowledge mobilization still falls outside the scope of most research funding. One finding that emerged through our policy engagement work on everyday risks was that there are often key policy champions that do demonstrate the will to address climate resilience; however, they often lack the capacity to understand and respond to this knowledge effectively. Building capacity and promoting learning requires additional funding^(97)^ and time.

Indeed, transformative resilience and the “triple-loop” learning it requires are long-term processes. However, researchers that embrace the action research approach can embed structures and processes in their projects that encourage ongoing learning. The UCRSEA partnership recognizes that, given the complex nature of urban climate resilience, the process of transformative resilience will outlast the duration of our project’s intervention. Therefore, the project sought to build skills for research and professional reflection among all of its partners through the various workshops and training activities we supported, to create a culture of critical reflection within these communities. Examples of this effort are the development of an urban climate resilience curriculum and an applied learning simulation methodology that researchers can adapt for use with local stakeholders, to continue to build knowledge, skills and intergroup empathy in ways that traditional researcher–policymaker interactions do not.

Together, such multi-pronged and long-term approaches allow for the centring of rights and justice in urban climate resilience. By creating knowledge through participatory citizen science and based on vulnerability to everyday risks, and by using knowledge to engage and motivate policymakers and to inform and empower urban residents, the UCRSEA project provides multiple examples of how transformative resilience work can be undertaken. While UCRSEA does not point to widespread societal change in any of the countries where the project worked – which would be unrealistic for a single research initiative – we argue that our overall approach can enable resilience scholarship to contribute to important changes over time. Most importantly, this work adds additional support to the call to inject rights and justice into climate resilience work in the cities where it is most urgently needed.

## Supplemental Material

sj-docx-1-eau-10.1177_09562478211035644 – Supplemental material for Rights, justice and climate resilience: lessons from fieldwork in urban Southeast Asia Click here for additional data file. Supplemental material, sj-docx-1-eau-10.1177_09562478211035644 for Rights, justice and climate resilience: lessons from fieldwork in urban Southeast Asia by Rebecca Mcmillan, Joanna Kocsis and Amrita Daniere in Environment & Urbanization

## Notes

1. Ziervogel et al. (2017), page 123.

2. Johannessen et al. (2018); Pahl-Wostl (2009); Pelling et al. (2008); Voß and Bornemann (2011).

3. For more details on the objectives, activities and accomplishments of the various partners please see http://ucrsea.ca.

4. Adger et al. (2005); Gunderson and Holling (2002).

5. Adger (2000).

6. Barnett and Bai (2007); Tyler and Moench (2012).

7. Boyd and Juhola (2015); da Silva et al. (2012).

8. Quay (2010); Ruth and Coelho (2007); Tyler and Moench (2012).

9. Folke et al. (2005); Lebel et al. (2006).

10. Bahadur and Tanner (2014); Moench et al. (2015); Reed et al. (2013).

11. Adger (2006).

12. Taylor (2013).

13. Cretney (2014).

14. Ziervogel et al. (2017).

15. MacKinnon and Derickson (2012); Peyroux (2015); Welsh (2014).

16. MacKinnon and Derickson (2012).

17. Pelling (2011).

18. Bahadur and Tanner (2014); O’Brien (2017); Pelling (2011); Pelling et al. (2015).

19. Ziervogel et al. (2017), page 135.

20. O’Brien and Selboe (2015); Paavola and Adger (2006).

21. Bulkeley et al. (2013).

22. Pelling (2011).

23. Solecki et al. (2017).

24. Argyris and Schön (1996); Pahl-Wostl (2009); Pelling et al. (2008); Wolfram et al. (2019).

25. Argyris and Schön (1996).

26. Wolfram et al. (2019), page 4.

27. Henrique and Tschakert (2019).

28. Tanner et al. (2009); UN-Habitat (2012).

29. Hijioka et al. (2014).

30. UNDP (2012). The UCRSEA project focused on secondary cities (cities of more than 50,000 people, excluding primary and/or capital cities) in the region because demographic trends indicate that these are the fastest-growing cities on a per-capita basis. They are also highly vulnerable to climate change impacts, but often lack the resources needed to effectively implement adaptation strategies. The cities included in the project are listed in Table 1 and illustrated in Map 1.

31. Dasgupta and Beard (2007); Lebel et al. (2011); Manuta et al. (2006).

32. Rittel and Webber (1973).

33. Turnbull and Hoppe (2019), page 333.

34. Denscombe (2010), page 126.

35. Somekh (1995), page 344.

36. Pahl-Wostl (2009).

37. Reed et al. (2013).

38. Ziervogel et al. (2017).

39. Kania and Kramer (2011).

40. Wolfram et al. (2019), page 4; Pelling et al. (2008).

41. Folke et al. (2005).

42. E.g. Ernstson et al. (2010).

43. Vogel (2012), page 5.

44. Mason and Barnes (2007).

45. Derbyshire (2018); Guarneros-Meza et al. (2018); Rolfe (2019).

46. Wolfram et al. (2019), page 5.

47. UCRSEA (2017).

48. See also Folke et al. (2005); Tyler and Moench (2012).

49. See in particular Daniere and Garschagen (2019); or visit http://ucrsea.ca.

50. While UCRSEA research teams’ specific methodologies and lenses were as diverse as their research contexts, they shared a commitment to bridging systems and actor-oriented approaches to resilience and vulnerability. Systems approaches to resilience emphasize how climate shocks interact with and cascade across interconnected urban systems at the city scale. [See e.g. Boyd and Juhola (2015); Burch et al. (2014); da Silva et al. (2012); Davidson and Venning (2011); Moench et al. (2015); Ruth and Coelho (2007); and Tyler and Moench (2012).] Meanwhile, actor-oriented approaches privilege bottom-up accounts of different groups’ capacities to confront change, as well as how they understand and perceive their own vulnerability and exert agency. [Martin et al. (2019); Turner (2012).]

51. Livelihoods approaches are commonly used in vulnerability research to understand how households’ capacities and assets (broadly defined) enable them to pursue their livelihoods and cope with shocks and stresses. For an overview of livelihoods frameworks see Ashley and Carney (1999); Bebbington (1999); Moser and Dani (2013); and Scoones (1998).

52. For more on UNISDR’s Disaster Resilience Scorecard for Cities, visit https://www.unisdr.org/campaign/resilientcities/toolkit/article/disaster-resilience-scorecard-for-cities. The Disaster Resilience Scorecard for Cities was previously called the Local Government Self-Assessment Tool. UNDRR is the current name of UNISDR.

53. Sudhipongpracha and Dahiya (2019).

54. Beringer and Kaewsuk (2018).

55. Marks (2019).

56. Beringer and Kaewsuk (2018).

57. This framework is rooted in resilience and systems thinking that conceives of climate change resilience as a product of the interaction of urban systems, agents and institutions.

58. Beringer and Kaewsuk (2018).

59. Reed et al. (2013).

60. Ziervogel et al. (2017), page 128.

61. Beringer and Kaewsuk (2018).

62. Martin et al. (2019).

63. Hoang and Pulliat (2019), page 180. Vietnam categorizes cities and towns according to population and economic status for the purpose of prioritizing planning activities and investments. A level 1 urban centre is the highest possible classification with the exception of the so-called centrally controlled cities, which include Hanoi and Ho Chi Minh City.

64. Nguyen Ngoc, Thanh Hoa and Ngoc Anh (2018), page 2.

65. Hoang and Pulliat (2019), page 186.

66. See reference 52.

67. Hoang and Pulliat (2019), page 186.

68. Hoang and Pulliat (2019).

69. See Folke et al. (2005); Lebel et al. (2006); Pahl-Wostl (2009); Quay (2010); Ruth and Coelho (2007).

70. Garschagen (2016).

71. See Marks and Lebel (2016).

72. Brown et al. (2012); de Souza et al. (2015).

73. Andrews et al. (2017).

74. Daniere and Garschagen (2019).

75. Nguyen Ngoc, Thanh Hoa, Ngoc Anh and Dang (2018); MSU (2017); Thuon and Heng (2018).

76. Denscombe (2010), page 128.

77. Earl et al. (2001).

78. Earl et al. (2001).

79. This strategy, labelled “positioning for use” in the International Development Research Centre’s “Research Quality Plus” research assessment framework, recognizes that *“[t]he uptake of research is inherently a political process. Preparing for it therefore requires attention to user contexts, accessibility of products, and ‘fit for purpose’ engagement and dissemination strategies.”* [Ofir et al. (2016), page 10.] In order to increase the likelihood of research uptake, positioning for use *“calls for strategies to integrate potential users into the research process itself wherever this is feasible and desirable”.* [Ofir et al. (2016), page 10.]

80. See Wolfram et al. (2019).

81. Wolfram et al. (2019).

82. Pelling et al. (2008).

83. Pahl-Wostl (2009).

84. Try Thuon, personal communication, 7 January 2021.

85. Beard (2003).

86. Beard (2003).

87. Beard (2003).

88. Denscombe (2010).

89. https://www.facebook.com/ucrsea/videos/in-this-video-cecr-and/1615639438510405.

90. http://ucrsea.ca/publications/citizen-science.

91. One of the most entertaining interventions involved creating a piece of theatre to share lessons learned using the AirBeam with volunteer health workers located in urban communities adjacent to Mahasarakham University, one of the partners that engaged fully with outreach in terms of using knowledge to connect with residents (https://drive.google.com/file/d/1FPbJN5G_fcix9kdgf0ZIiZZWk8rqzXUF/view).

92. Liobikien and Poškus (2019).

93. Simpson and Smits (2018).

94. See Daniere and Garschagen (2019).

95. Ziervogel et al. (2017).

96. Hallegatte and Mach (2016).

97. See CARIAA (2018).
