# Supplementary material for: Rights, justice and climate resilience: lessons from fieldwork in urban Southeast Asia
Source: Environ Urban. 2021 Aug 22;34(1):170–89. doi: 10.1177/09562478211035644 (PMC9047090; doi:10.1177/09562478211035644)
Supplement: sj-docx-1-eau-10.1177_09562478211035644 – Supplemental material for Rights, justice and climate resilience: lessons from fieldwork in urban Southeast Asia [file sj-docx-1-eau-10.1177_09562478211035644.docx]

**Rights, justice and climate resilience: lessons from fieldwork in urban Southeast Asia**

Online supplementary information

**List of UCRSEA partner**s

Center for Environment and Community Research, Vietnam

Center for Natural Resources and Environmental Studies, Vietnam National University, Vietnam

Center for Peace and Conflict Studies, Chulalongkorn University, Thailand

Faculty of Development Studies, Royal University of Phnom Penh, Cambodia

Faculty of Environment and Resource Studies, Mahasarakham University, Thailand

General Department of Administration for Nature Conservation and Protection, Ministry of the Environment, Cambodia

Mercy Corps Myanmar

Regional Center for Social Science and Sustainable Development, Faculty of Social Sciences, Chiang Mai University, Thailand

Renewable Energy Association Myanmar

Research Group on Wellbeing and Sustainable Development, Khon Kaen University, Thailand

Swanyee Development Foundation, Myanmar

Thailand Environment Institute, Thailand

University of Ottawa, Canada

University of Toronto, Canada

University of Yangon, Myanmar

York University, Canada
